# Antimicrobial Potential of Endophytic Fungi Derived from Three Seagrass Species: *Cymodocea* *serrulata*, *Halophila* *ovalis* and *Thalassia* *hemprichii*

**DOI:** 10.1371/journal.pone.0072520

**Published:** 2013-08-16

**Authors:** Preuttiporn Supaphon, Souwalak Phongpaichit, Vatcharin Rukachaisirikul, Jariya Sakayaroj

**Affiliations:** 1 Department of Microbiology and Natural Products Research Center of Excellence, Faculty of Science, Prince of Songkla University, Hat Yai, Songkhla, Thailand; 2 Department of Chemistry and Center of Excellence for Innovation in Chemistry Faculty of Science, Prince of Songkla University, Hat Yai, Songkhla, Thailand; 3 National Center for Genetic Engineering and Biotechnology, Klong Luang, Pathumthani, Thailand; Broad Institute of Harvard and MIT, United States of America

## Abstract

Endophytic fungi from three commonly found seagrasses in southern Thailand were explored for their ability to produce antimicrobial metabolites. One hundred and sixty endophytic fungi derived from *Cymodocea* *serrulata* (Family Cymodoceaceae), *Halophila* *ovalis* and *Thalassia* *hemprichii* (Family Hydrocharitaceae) were screened for production of antimicrobial compounds by a colorimetric broth microdilution test against ten human pathogenic microorganisms including *Staphylococcus aureus* ATCC 25923, a clinical isolate of methicillin-resistant *S. aureus*, *Escherichia coli* ATCC 25923, *Pseudomonas aeruginosa* ATCC 27853, *Candida albicans* ATCC 90028 and NCPF 3153, *Cryptococcus neoformans* ATCC 90112 and ATCC 90113 and clinical isolates of *Microsporum* *gypseum* and *Penicillium* *marneffei* . Sixty-nine percent of the isolates exhibited antimicrobial activity against at least one test strain. Antifungal activity was more pronounced than antibacterial activity. Among the active fungi, seven isolates including Hypocreales sp. PSU-ES26 from *C* *. serrulata* , *Trichoderma* spp. PSU-ES8 and PSU-ES38 from *H* *. ovalis* , and *Penicillium* sp. PSU-ES43, *Fusarium* sp. PSU-ES73, *Stephanonectria* sp. PSU-ES172 and an unidentified endophyte PSU-ES190 from *T* *. hemprichii* exhibited strong antimicrobial activity against human pathogens with minimum inhibitory concentrations (MIC) of less than 10 µg/ml. The inhibitory extracts at concentrations of 4 times their MIC destroyed the targeted cells as observed by scanning electron microscopy. These results showed the antimicrobial potential of extracts from endophytic fungi from seagrasses.

## Introduction

Endophytic fungi are fungi found growing in healthy plants within various tissues [1]. They associate with their host plant, often produce substances that can help to protect host plants from insect and pathogenic microorganisms and enhance the growth rate of their hosts. It has been reported that 51% of all biologically active substances have been isolated from endophytic fungi [2]. Since the discovery of taxol, a powerful anticancer agent from the bark of *Taxus* *brevifolia* (Pacific Yew) and from endophytic fungi from *Taxus* spp. [3], endophytic fungi from various plants that are claimed to have medicinal properties have been investigated and shown to produce bioactive metabolites including anticancer, antibacterial, antifungal, anti-malarial and enzyme activities [4–6].

Antimicrobial natural products from plants have been extensively studied in the hope they might inhibit the growth of the many new antibiotic resistant pathogens that have recently emerged. The expectations of these new inhibitory agents from natural products are that they are effective, have low toxicity and have a low environmental impact [1]. Most endophytic fungi have been isolated from terrestrial plants including dicotyledons and monocotyledons [7]. Seagrasses are flowering plants in the class Monocotyledons that evolved from terrestrial plants millions of years ago. However, endophytic fungi from seagrasses have been rarely studied [8–10]. The coastal areas of Trang Province in southern Thailand have many rich seagrass beds [11]. Recently, Arunpanichlert et al. [12,13] reported new and bioactive metabolites isolated from endophytic fungi such as by *Fusarium* sp. PSU-ES73 isolated from *Thalassia* *hemprichii* and *Bipolaris* sp. PSU-ES64 from *Halophila* *ovalis* both from Thailand. This study reports on the isolation of endophytic fungi from three of the dominant seagrasses; *Cymodocea* *serrulata* , *H* *. ovalis* and *T* *. hemprichii* from Trang Province, their ability to produce antimicrobial metabolites against ten human pathogens and the possible mechanism of antimicrobial actions of the most active extracts as revealed by scanning electron microscopy (SEM). The identity of the isolates producing these inhibitory compounds was also investigated by observing their morphology and by molecular characterisation.

**Table 1:** Number of active isolates and active extracts from endophytic fungi from three seagrass species.

		*Cymodocea*	*Halophila*		*Thalassia*	Total
		*serrulata*	*Ovalis*		*hemprichii*
No. of active isolates/		9/12	53/69		48/79	110/160
Total no. of isolates tested (%)		(75.0)	(76.8)		(60.8)	(68.8)
No. of active extracts/		19/34	94/199		96/224	209/457
Total no. of extracts tested (%)		(55.9)	(47.2)		(42.9)	(45.7)

## Materials and Methods

### Seagrass sample collection and isolation of endophytic fungi

Healthy seagrass samples were collected monthly from June 2008 to May 2009 from Pak Meng beach, Trang Province, Thailand. No specific permissions were required for this location because it was not part of a national park and the field studies did not involve endangered or protected species. The samples were identified as *C* *. serrulata* , *H* *. ovalis* and *T* *. hemprichii* according to their morphologies [14,15]. Seagrass leaves, roots and rhizomes were washed thoroughly by running tap water and surface sterilized in 10% ethanol for 3 min, 3% sodium hypochlorite for 1 min, 10% ethanol for 3 min and rinsed twice in sterile distilled water. Samples were blotted dry with sterile paper towels, then cut into 6 small segments and placed on potato dextrose-seawater agar (PDA-SW) containing 50 mg/l penicillin and streptomycin. Plates were incubated at 25°C until the outgrowth of endophytic fungi was observed, then the outgrowths were subcultured to produce pure culture on PDA-SW without antibiotics by the hyphal tip isolation method. All isolates were maintained in 20% glycerol at -80^o^C.

### Fungal identification

All active fungal isolates were identified initially by their morphological characteristics. Those isolates that produced no reproductive structure were identified only by molecular techniques based on the analyses of their nuclear ribosomal internal transcribed spacer (ITS) regions.

Fungal mycelia grown in potato dextrose broth (PDB) for one week were collected by filtering through sterile cheesecloth and washing with warm sterile distilled water twice. Fungal genomic DNA was extracted using the DNeasy Plant Mini Kit (Qiagen) following manufacturer’s instruction. Primer pairs ITS1F/ITS4, ITS1/ITS4 and ITS5/ITS3 were used for the PCR reactions [16,17]. The purified PCR-DNA products were sequenced by Macrogen, a Korean biotechnology company using the same primers. The sequences were analyzed by BioEdit 7.0.7 [18]. The sequences were then compared with the GenBank database by the BLASTN program. Phylogenetic relationships were estimated using PAUP* v4.0b10 [19]. DNA sequences of our active isolates have been submitted to NCBI GenBank database for retrieval of accession numbers.

### Broth fermentations and extractions

Endophytic fungi were cultured in PDB at 25^o^C for 3-4 weeks under stationary conditions as previously described [20]. Fermentation broths were separated from the mycelium by filtration. The culture filtrates were extracted twice with an equal volume of ethyl acetate (EtOAc) in a separating funnel. The ethyl acetate layers were evaporated to dryness under reduced pressure at 40-45°C using a rotary evaporator to obtain a crude broth ethyl acetate extract (BE). The fungal mycelia were soaked in methanol for 2 days. The methanol layer was re-extracted with hexane, then again with ethyl acetate. The hexane and the ethyl acetate extracts of the mycelia were evaporated to dryness to give the cell hexane (CH) and cell ethyl acetate (CE) extracts, respectively. The dry crude extracts were dissolved with dimethylsulfoxide (DMSO) to prepare stock solutions of 10 mg/ml and kept for antimicrobial testing.

### Antimicrobial screening

The following test microorganisms were used for screening of antimicrobial activities: *Staphylococcus aureus* ATCC 25923, a clinical isolate of methicillin-resistant *S. aureus* (MRSA), *Escherichia coli* ATCC 25922, *Pseudomonas aeruginosa* ATCC 27853, *Candida albicans* ATCC 90028 and NCPF 3153, *Cryptococcus neoformans* ATCC 90112 (flucytosine-sensitive) and ATCC 90113 (flucytosine-resistant), clinical isolates of *Microsporum* *gypseum* and *Penicillium* *marneffei* . Fungal extracts at final concentrations of 200 µg/ml were preliminarily tested against all the test microorganisms by a colorimetric broth microdilution method in 96-well microtiter plates according to the Clinical and Laboratory Standards Institute [21–23] with some modifications. The test microtiter plates were incubated at 35°C for 15 h for bacteria and *C. albicans*, 25°C for 45 h for *C. neoformans*, 25°C for 6 days for *M* *. gypseum* , then 10 µl of a 0.18% resazurin solution was added into each well and further incubated for another 2-3 h for bacteria and yeasts and 1 day for fungi as adapted from Sarker et al. [24]. Vancomycin, gentamicin, amphotericin B and miconazole were used as positive controls for the Gram-positive bacteria, Gram-negative bacteria, yeasts and fungi, respectively. The color change was then observed visually. Any color changes from purple to pink or colorless were recorded as negative. A blue or purple color of the wells indicated inhibition of growth (positive result). *P* *. marneffei* was tested in the same manner as *M* *. gypseum* but no resazurin was added because of its red pigment production. Therefore, its growth was observed using a stereomicroscope.

### Determination of minimum inhibitory concentration (MIC), minimum bactericidal concentration (MBC) and minimum fungicidal concentration (MFC)

The crude extracts that showed antimicrobial activity at 200 µg/ml were further assessed for their MICs, MBCs or MFCs by the same method using 2-fold serially diluted crude extracts from 0.25 to 128 µg/ml. The lowest concentration of extract that inhibited growth was recorded as the MIC. All positive wells that showed growth inhibition were streaked onto nutrient agar for bacteria or Sabouraud dextrose agar for yeasts and fungi and incubated under appropriate conditions. The lowest concentration of extract that exhibited no visible growth was considered to be the MBC or MFC. MIC activities recognized as strong were <10 µg/ml.

### Scanning electron microscopic analysis

Extracts with strong antimicrobial activity were further investigated for their possible effects on targeted cells by SEM. Test organism treated with crude extract at 4 times their MIC values for 18 h was fixed with 2.5% glutaraldehyde (C_3_ H_8_ O_2_) in phosphate buffer solution (PBS) for 2 h, then washed with PBS and water, post fixed in 1% osmium tetroxide for 1 h and washed with water. The samples were dehydrated with alcohol series (50%, 70%, 80%, 90% and 100% ethanol). Samples dried using a critical point dryer (Polarum, CP07501), were sputtered with gold and scanned under SEM (Model JSM-5800 LV, Type LV, JEOL Ltd., Japan) at the Scientific Equipment Center, Prince of Songkla University.

## Results

### Isolation of endophytic fungi

One hundred and sixty endophytic fungal isolates were obtained from 4,980 tissue segments of the leaf, root and rhizome of *C* *. serrulata* , *H* *. ovalis* and *T* *. hemprichii* . The overall isolation rate was 3.2%. Most of the isolated endophytic fungi (95%) were from the leaf segments.

### Antimicrobial screening

Fungal crude extracts at concentrations of 200 µg/ml were screened for antibacterial activity against Gram-positive bacteria ( *S* *. aueus* ATCC 25923 and MRSA clinical isolate), Gram-negative bacteria (*E. coli* ATCC 25923 and *P. aeruginosa* ATCC 27853), pathogenic yeasts (*C. albicans* ATCC 90028 and NCPF 3153; and *C. neoformans* ATCC 90112 and ATCC 90113), and pathogenic filamentous fungi ( *M* *. gypseum* and *P* *. marneffei* clinical isolates). Two hundred and nine extracts out of 457 tested extracts (45.7%) from 110 isolates (68.8%) were active against one to eight test strains. The percentage of active isolates from each host plant species including *C* *. serrulata* , *H* *. ovalis* and *T* *. hemprichii* was significant at 75.0%, 76.8% and 60.8%, respectively (Table 1).

**Figure 1:**
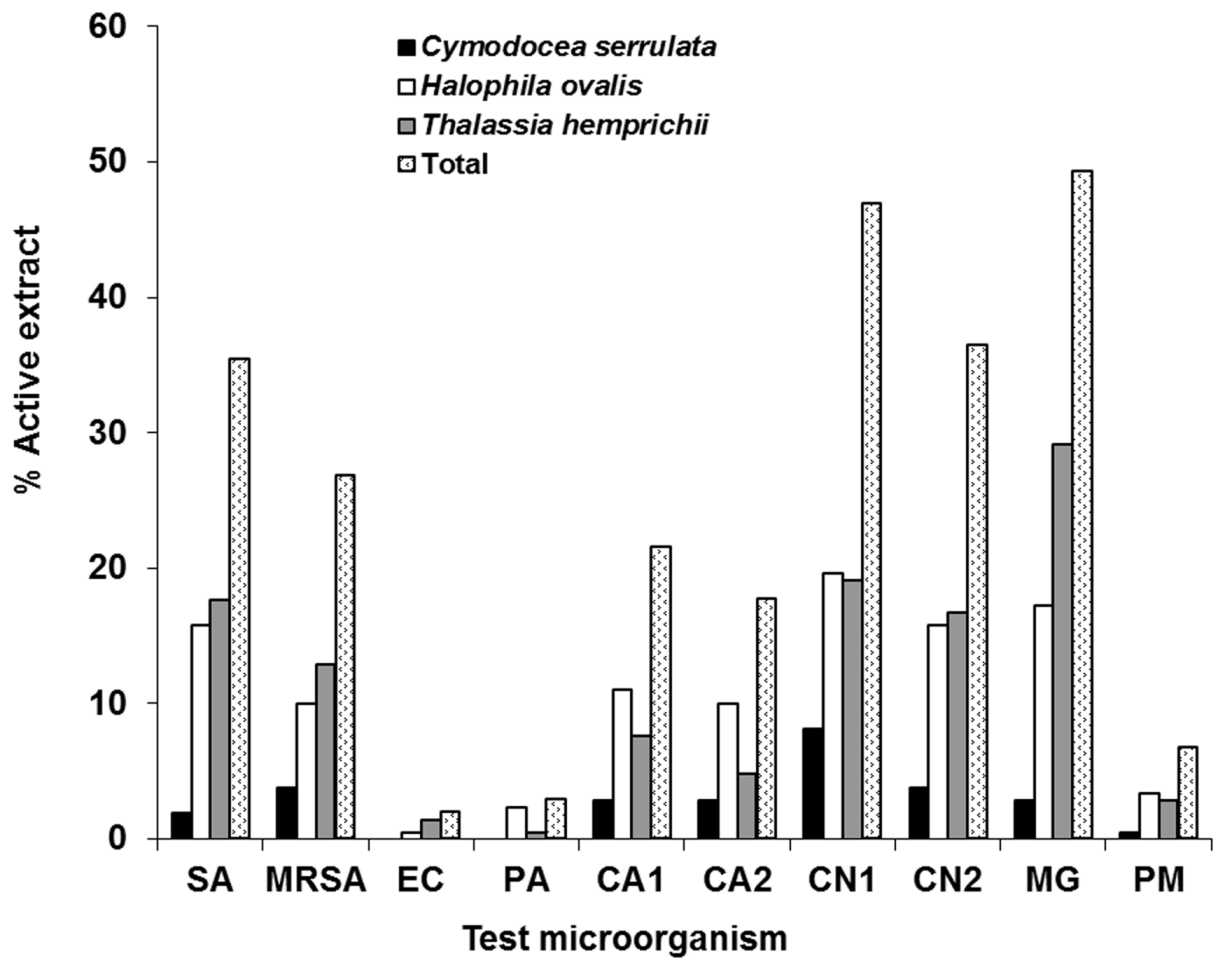
Antimicrobial activity of endophytic fungal crude extracts against each test microorganism. SA, *Staphylococcus aureus* ATCC 25923; MRSA, methicillin-resistant *S. aureus*; EC, *Escherichia coli* ATCC 25922; PA, *Pseudomonas aeruginosa* ATCC 27853; CA1, *Candida albicans* ATCC 90028; CA2, *C. albicans* NCPF 3153; CN1, *Cryptococcus neoformans* ATCC 90112 (flucytosine-sensitive); CN2, *C. neoformans* ATCC 90113 (flucytosine-resistant); MG, *Microsporum* *gypseum* clinical isolate; PM, *Penicillium* *marneffei* clinical isolate.

The endophytic fungal extracts were most active against *M* *. gypseum* (49%) followed by both strains of *C. neoformans* (36-47%), then *S. aureus* (27-35%), *C. albicans* (18-22%), *P* *. marneffei* (7%), *P. aeruginosa* (3%) and *E. coli* (2%). Most of the active fungal extracts were derived from *T* *. hemprichii* (Figure 1). The majority of the extracts had a narrow spectrum of activity against only bacteria, yeasts or fungi. Only 28.3% had a broad spectrum activity against both bacteria and fungi (Table 2).

### Determination of minimum inhibitory concentration (MIC), minimum bactericidal concentration (MBC) and minimum fungicidal concentration (MFC)

All active extracts were further determined for their MIC, MBC or MFC by a colorimetric microdilution test. The MIC values of all 209 active extracts ranged from 1 to 200 µg/ml. Eight extracts from seven isolates showed strong antimicrobial activities with MIC values less than 10 µg/ml (Table 3). Cell hexane extract (CH) of PSU-ES26 yielded the best antifungal activity against *M* *. gypseum* (MIC/MFC 2/16 µg/ml). This was comparable to the antifungal drug miconazole. It also showed good inhibition of *C. albicans* and *C. neoformans*. Both cell ethyl acetate extracts (CE) from PSU-ES8 and PSU-ES38 strongly inhibited *C. albicans* NCPF 3153 (MIC 1-2 µg/ml). Extracts from PSU-ES43, PSU-ES73 and PSU-ES190 mostly inhibited *C. neoformans*. Among them, the CE extract of PSU-ES73 was the best against *C. neoformans* ATCC 90112 (MIC/MFC 1/4 µg/ml) and ATCC 90113 (MIC/MFC 4/8 µg/ml). It also showed strong activity against both strains of *S. aureus*. The broth ethyl acetate (BE) and CE extracts from PSU-ES172 had strong activity only against *S. aureus* ATCC 25923 (MIC 8 µg/ml). There was no extract that showed strong activity against *E. coli*, *P. aeruginosa* and *P* *. marneffei* .

### Scanning electron microscopic (SEM) analysis

**Figure 2:**
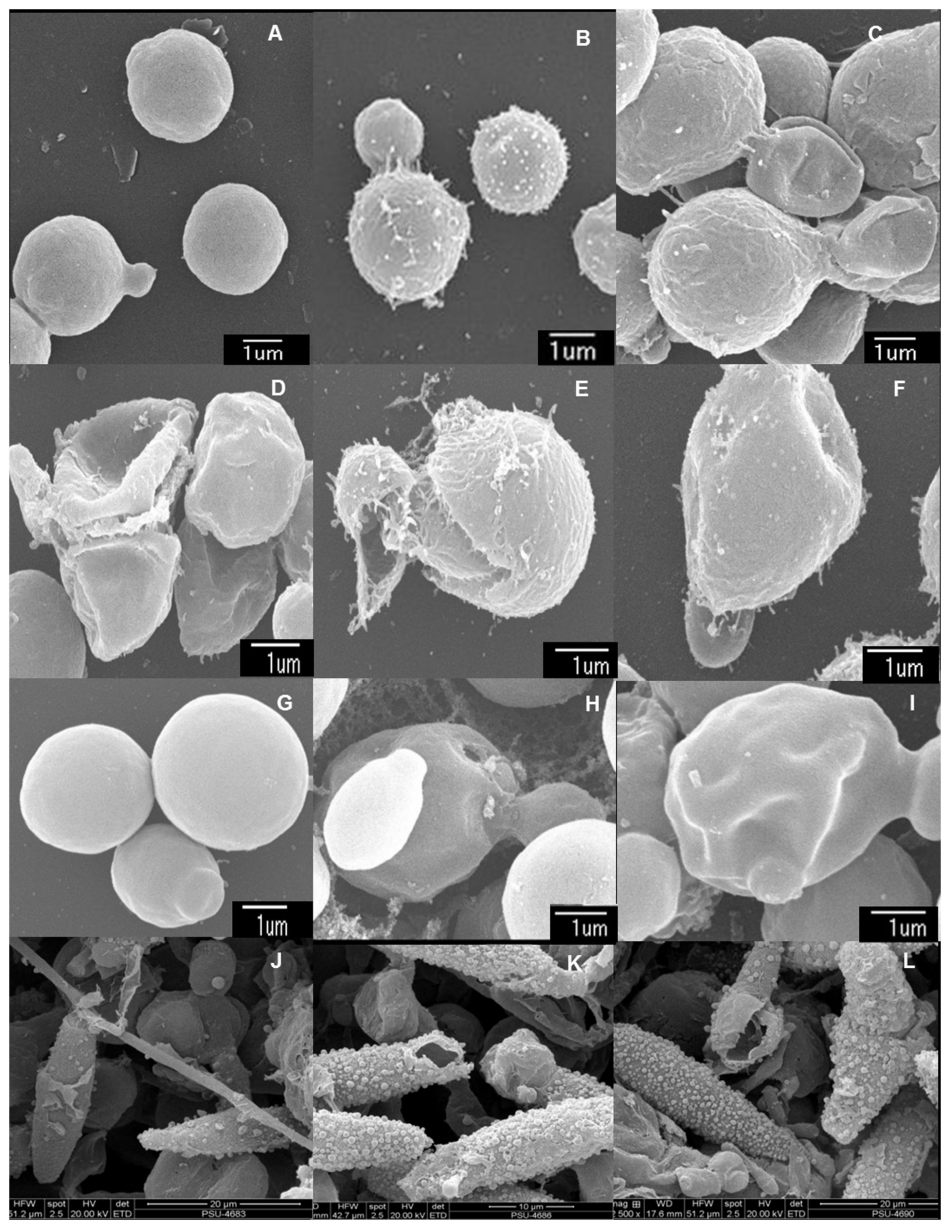
Scanning electron micrographs of test microorganisms with strongly active crude extracts. *Cryptococcus neoformans* ATCC 90112 (A–E), *Cryptococcus neoformans* ATCC 90113 (F), *Candida albicans* NCPF 3153 (G–I) and a clinical isolate of *Microsporum* *gypseum* (K–L) after incubation with 10% DMSO (A, G and J), amphotericin B (B and H), miconazole (K), hexane extract from the mycelia of *Penicillium* sp. PSU-ES43 (C), hexane extract from the mycelia of PSU-ES190 (D), ethyl acetate extract from the mycelia of *Fusarium* sp. PSU-ES73 (E and F), ethyl acetate extract from the mycelia of *Trichoderma* sp. PSU-ES38 (I), and hexane extract from the mycelia of Hypocreales sp. PSU-ES26 (L) for 16 h at 4 times their MIC values.

The effects of the most active crude extracts against their susceptible test microorganisms were investigated by SEM (Figure 2). SEM images of the targeted cells; *C. neoformans*, *C. albicans* and *M* *. gypseum* revealed ultrastructural changes due to the active extracts and standard antifungal agents. Control yeast cell treated with DMSO had well defined, intact shapes with smooth surfaces (Figure 2A and G). *C. neoformans* treated with x4 MIC concentrations of PSU-ES43CH (Figure 2C), PSU-ES190CH (Figure 2D), and PSU-ES73CE (Figure 2E and F) showed considerable morphological alterations including deformation, shrinkage, collapsed and broken cells. In amphotericin B treated cells, rough surfaces were noticed (Figure 2B). Similarly, cell wall invaginations were observed in *C. albicans* treated with x4 MIC of PSU-ES38CE (Figure 2I), whereas perforation was noticed in the cell surface of amphotericin B treated cells (Figure 2H). For *M* *. gypseum* , broken cells were observed in both samples treated with P (Figure 2K) and miconazole (Figure 2L) as compared to normal DMSO-treated cells (Figure 2J). The surface roughness of the treated cells was also increased. The active extracts apparently affect the fungal cell wall as well as cell membrane.

**Table 2:** Distribution of the antimicrobial spectrum of inhibitory endophytic fungal extracts.

**% Active**	**Activity**						
**Extracts**	**Antibacterial**	**Anti-yeast**	**Anti-filamentous fungi**
14.3	←--------------→	
19.1		←--------→
21.5			←--------------------------→		
16.8		←------------------------------------------------------------→		
14.4	←-------------------------------------------------→		
1.0	←-------------→		←--------------------------→		
12.9	←-----------------------------------------------------------------------------------------------------→							

### Identification of endophytic fungi with strong antimicrobial activity

**Figure 3:**
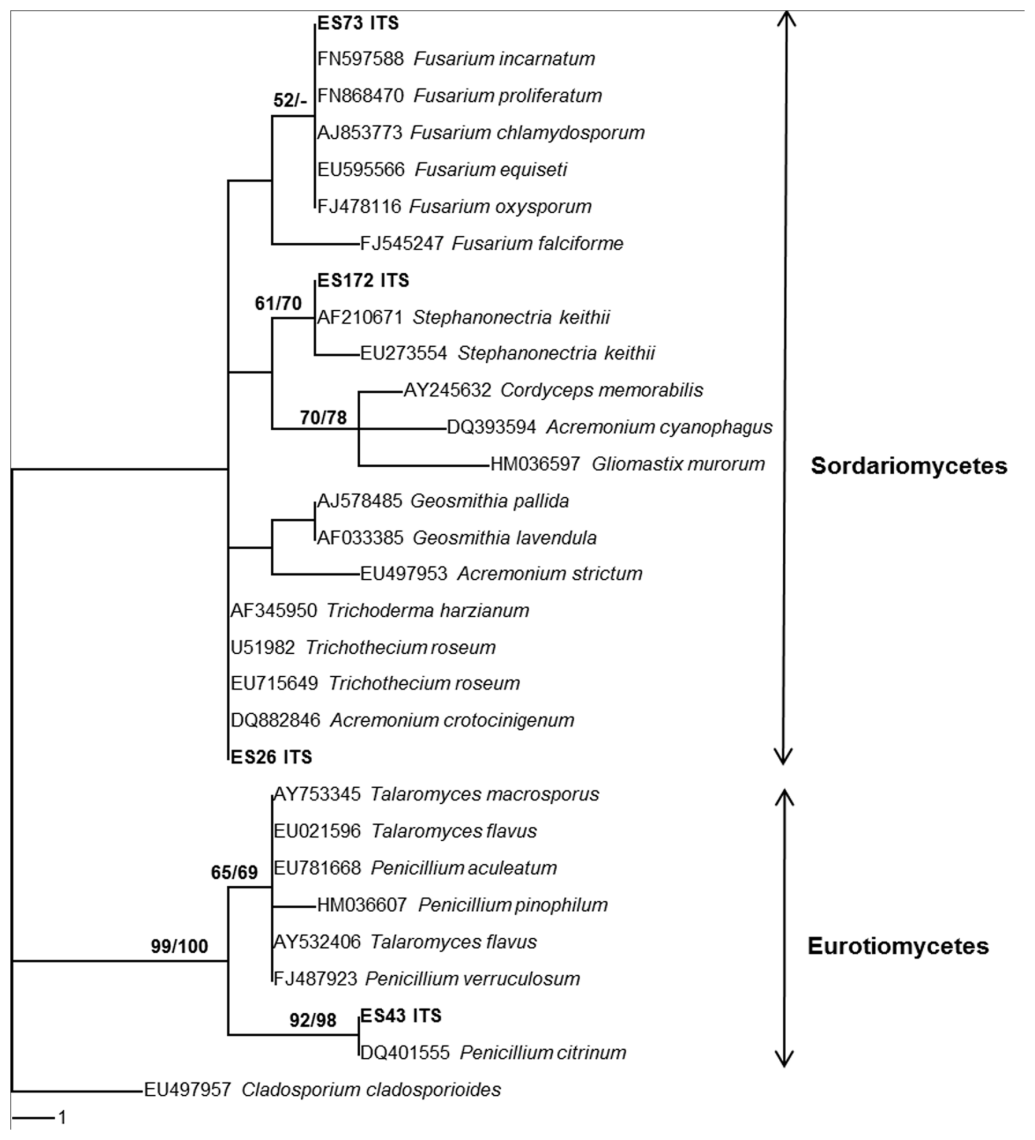
Phylogenetic tree based on ITS1-5.8S-ITS2 sequences of strongly active endophytic fungi. The number of each branch point represents percentage bootstrap support from Maximum Parsimony (MP BS) and Neighbour Joining (NJ BS) with 100 replications shown on the branch. MP BS values ≥50% are shown before the slash; NJ BS values ≥50% are shown after the slash. Length; 37 steps; consistency index (CI); 0.8108; retention index (RI); 0.9391; homoplasy index (HI); 0.1892; rescaled consistency index (RC); 0.7615.

The seven strongly active fungal isolates were PSU-ES26 from *C* *. serrulata* , PSU-ES8 and PSU-ES38 from *H* *. ovalis* and PSU-ES43, PSU-ES73 and PSU-ES172 from *T* *. hemprichii* . PSU-ES8 and PSU-ES38 produced scattered blue-green mass of conidia with micro-morphological characteristics of the genus *Trichoderma* . The rest did not sporulate in culture and were identified by molecular characterisation (Figure 3). PSU-ES43, PSU-ES73 and PSU-ES172 were identified to be in the genera *Penicillium* , *Fusarium* and *Stephanonectria*, respectively. While PSU-ES26 could be identified as a member of the Hypocreales sp. The ITS region of PSU-ES190 could not be amplified, therefore it was named as mycelia sterilia PSU-ES190.

**Table 3:** The endophytic fungal isolates from three species of seagrasses with strong antimicrobial activity.

**Seagrass**	^a)^ **Crude extract** (**Fungal identification**)	**Accession no.**						^b)^ **MIC /MBC or MFC values** (**µg/ml**)										
								^c)^ **SA**	**MRSA**	**CA1**	**CA2**	**CN1**	**CN2**	**MG**
** *Cymodocea* *serrulata* **				
PSU-ES26CH (Hypocreales sp.)			JN116602							**8**/128	**8**/128	16/>200	**8**/128	**2**/16
** *Halophila* *ovalis* **				
PSU-ES8CE ( *Trichoderma* sp.)^d^								64/>200		128/>200	**2**/>200			
PSU-ES38CE ( *Trichoderma* sp.)^d^								64/>200			**1**/>200			
** *Thalassia* *hemprichii* **				
PSU-ES43CH ( *Penicillium* sp.)			JN116612									**8/16**	32/64	
PSU-ES73CE ( *Fusarium* sp.)			JQ733502					**8**/>200	**8**/>200			**¼**	**4/8**	
PSU-ES172BE ( *Stephanonectria* sp.)			JN116689					**8**/>200							
PSU-ES172CE ( *Stephanonectria* sp.)			JN116689					**8**/>200							
PSU-ES190CH (mycelia sterilia)												**4**/>200	32/64	
Antibiotic drugs				
Vancomycin								0.5/1	1/2					
Amphotericin B										0.125/0.25	0.125/0.5	0.125/1	0.25/2	
Miconazole														1/32

^a)^CH, hexane extract from fungal cell; BE, ethyl acetate extract from culture broth; CE, ethyl acetate extract from fungal cell

^b)^MIC, minimum inhibitory concentration; MBC minimum bactericidal concentration; MFC, minimum fungicidal concentration

^c)^SA, *Staphylococcus aureus* ATCC 25923; CA1, *Candida albicans* ATCC 90028; CA2, *C. albicans* NCPF 3153; CN1, *Cryptococcus neoformans* ATCC 90112 (flucytosine-sensitive); CN2, *C. neoformans* ATCC 90113 (flucytosine-resistant); MG, *Microsporum* *gypseum* clinical isolate; PM, *Penicillium* *marneffei* clinical isolate

^d)^Identified by morphological characteristics

## Discussion

Infections caused by drug resistant microorganisms are increasing worldwide. In addition, many side effects of commonly used antifungal drugs have been reported. Amphotericin B derived from *Streptomyces* *nodosus* has been used to treat systemic fungal infections. It is well known for its severe and potentially lethal side effects including high fever and shaking chills after a few hours of infusion. Azole antifungal agents are widely used to treat various fungal diseases. Their antifungal mechanisms include inhibiting the synthesis of membrane ergosterol and this prevents fungal growth through inhibition of CYP450. These drugs can bind with human membranes, and damage kidneys [25]. Their general side effects include gastrointestinal disturbance, hepatotoxicity and a rash [26]. Therefore, there is a need to find new drugs from various sources. Sources other than terrestrial have been investigated for antimicrobial activities including a few marine sources. Nowadays, a search for novel bioactive metabolites from marine natural products is increasing because of their uniqueness [27].

Some of the seagrasses have been used in traditional medicine for example in India for malaria, skin diseases and the early stage of leprosy [28]. Some extracts also have antibacterial activity [28–33]. During the long period of co-evolution, a cooperative relationship has been formed between each endophyte and its host plant. Some endophytes have the ability to produce similar bioactive compounds to those that originate from their terrestrial host plants [34]. In this study, we isolated many endophytic fungi from three seagrass species commonly found in the south of Thailand and screened them for their ability to produce antimicrobial metabolites. Although low colonization densities of endophytic fungi have been reported in seagrasses [35], the percentage of active isolates derived from seagrasses (69%) was in the same range as those derived from mangrove plants (61%) [36] or even higher than those isolated from other terrestrial plants such as *Garcinia* species [20]. The number of active extracts and active isolates among the three studied seagrasses was similar. This indicated that these seagrasses are a good source of antimicrobial-producing endophytic fungi. The strongly active genera found in this study were *Fusarium* *,* *Penicillium* , *Stephanonectria* and *Trichoderma* . Most of them have been reported from both terrestrial and marine origins.

More than 50% of the fungal extracts had antifungal activity either against only pathogenic yeasts or only filamentous fungi or both. CH extracts from *Fusarium* sp. PSU-ES73 showed strong fungicidal activity against both strains of *C. neoformans* with MIC and MFC difference of 2 to 4 folds. Therefore, at 4 times its MIC it killed *C. neoformans* by destroying the cell surface. Arunpanichlert et al. [12] have isolated many pure compounds from this isolate including a new beta-resorcylic macrolide (5’ hydroxyzearalenone) and six known beta-resorcylic macrolides (zearalenone, 8'-hydroxyzearalenone, 7'-dehydrozearalenone, beta-zearalenol, 5'-hydroxyzearalenol and relgro). Only zearalenone showed moderate activity against *C. neoformans* (MIC 16 µg/ml). The crude extract was more active than the pure compound and that may result from synergistic effects of other constituents in the extract.

The CH extract from Hypocreales sp. PSU-ES26 showed very broad and strong antifungal activity against all the fungi tested except for *P* *. marneffei* . It destroyed *M* *. gypseum* macroconidia in the same manner as shown by miconazole. The active compounds from this isolate will be further investigated to determine their chemical identities.

Two species of *Trichoderma* (PSU-ES8 and PSU-ES38) isolated from *H* *. ovalis* had strong antifungal activity and moderate antibacterial activity. *Trichoderma* spp. are well-known as antifungal producers [37]. They have been extensively used as biological control agents for various phytopathogenic fungi. Vincente et al. [38] reported that ergokonin A produced by *T* *. longibrachiatum* exhibited activity against *Candida* and *Aspergillus* species but was inactive against *C. neoformans*. Extracts from our two *Trichoderma* spp. strongly inhibited *C. albicans* but had no activity against *C. neoformans*. These extracts may contain ergokonin A. Further investigations will be carried out.

The CH extract from *Penicillium* sp. PSU-ES43 exhibited strong to moderate activity only against both strains of *C. neoformans*. *Penicillium* spp. have been most studied in the bioperspective program since the discovery of penicillin. They produce various kinds of secondary metabolites including antibacterial [39,40] and antifungal substances [41–43]. Most of those reported isolates were from soils. Arunpanichlert et al. [44] reported that sclerotiorin isolated from the endophytic fungus *Penicillium* *sclerotiorum* PSU-A13 had moderate antifungal activity against *C. albicans* and *C. neoformans*. In contrast Trisuwan et al. [45] reported nine new compounds from a sea fan-derived *Penicillium* sp. PSU-F40. Some of those compounds exhibited antibacterial activity. The active metabolites from our *Penicillium* sp. PSU-ES43 will be further studied.

In this study, we screened a large number of endophytic fungi from three seagrass species and found a high percentage of isolates that produced active compounds. Some of them showed strong antifungal activity that had MIC values close to the antifungal drug and this was confirmed by the SEM study. This finding confirms the potential for investigating endophytic fungi from marine plants.
